# Protective Effects of Hepatocyte Stress Defenders, Nrf1 and Nrf2, against MASLD Progression

**DOI:** 10.3390/ijms25158046

**Published:** 2024-07-24

**Authors:** May G. Akl, Lei Li, Scott B. Widenmaier

**Affiliations:** Department of Anatomy, Physiology, and Pharmacology, University of Saskatchewan, Saskatoon, SK S7N 5E5, Canada; mgm228@usask.ca (M.G.A.);

**Keywords:** MASLD, MASH, liver fibrosis, HCC, Nrf1, Nrf2

## Abstract

Progression of metabolic dysfunction-associated steatites liver disease (MASLD) to steatohepatitis (MASH) is driven by stress-inducing lipids that promote liver inflammation and fibrosis, and MASH can lead to cirrhosis and hepatocellular carcinoma. Previously, we showed coordinated defenses regulated by transcription factors, nuclear factor erythroid 2-related factor-1 (Nrf1) and -2 (Nrf2), protect against hepatic lipid stress. Here, we investigated protective effects of hepatocyte Nrf1 and Nrf2 against MASH-linked liver fibrosis and tumorigenesis. Male and female mice with flox alleles for genes encoding Nrf1 (*Nfe2l1*), Nrf2 (*Nfe2l2*), or both were fed a MASH-inducing diet enriched with high fat, fructose, and cholesterol (HFFC) or a control diet for 24–52 weeks. During this period, hepatocyte Nrf1, Nrf2, or combined deficiency for ~7 days, ~7 weeks, and ~35 weeks was induced by administering mice hepatocyte-targeting adeno-associated virus (AAV) expressing Cre recombinase. The effects on MASH, markers of liver fibrosis and proliferation, and liver tumorigenesis were compared to control mice receiving AAV-expressing green fluorescent protein. Also, to assess the impact of Nrf1 and Nrf2 induction on liver fibrosis, HFFC diet-fed C57bl/6J mice received weekly injections of carbon tetrachloride, and from week 16 to 24, mice were treated with the Nrf2-activating drug bardoxolone, hepatocyte overexpression of human NRF1 (hNRF1), or both, and these groups were compared to control. Compared to the control diet, 24-week feeding with the HFFC diet increased bodyweight as well as liver weight, steatosis, and inflammation. It also increased hepatocyte proliferation and a marker of liver damage, p62. Hepatocyte Nrf1 and combined deficiency increased liver steatosis in control diet-fed but not HFFC diet-fed mice, and increased liver inflammation under both diet conditions. Hepatocyte Nrf1 deficiency also increased hepatocyte proliferation, whereas combined deficiency did not, and this also occurred for p62 level in control diet-fed conditions. In 52-week HFFC diet-fed mice, 35 weeks of hepatocyte Nrf1 deficiency, but not combined deficiency, resulted in more liver tumors in male mice, but not in female mice. In contrast, hepatocyte Nrf2 deficiency had no effect on any of these parameters. However, in the 15-week CCL4-exposed and 24-week HFFC diet-fed mice, Nrf2 induction with bardoxolone reduced liver steatosis, inflammation, fibrosis, and proliferation. Induction of hepatic Nrf1 activity with hNRF1 enhanced the effect of bardoxolone on steatosis and may have stimulated liver progenitor cells. Physiologic Nrf1 delays MASLD progression, Nrf2 induction alleviates MASH, and combined enhancement synergistically protects against steatosis and may facilitate liver repair.

## 1. Introduction

Metabolic dysfunction-associated steatotic liver disease (MASLD; also known as non-alcoholic fatty liver disease) is prevalent in 37% of the global population and has a wide clinicopathological spectrum [1,2,3]. The most common case is asymptomatic simple steatosis, while ~20% of people with MASLD progress to metabolic dysfunction-associated steatohepatitis (MASH), characterized by liver steatosis, inflammation, and fibrosis. People with MASH have higher risk for cirrhosis and hepatocellular carcinoma (HCC), and so treatments that delay or reverse MASH onset and its progression to more severe disease stages are in need [4,5,6,7,8]. A major factor driving MASH is the accumulation of hepatotoxic lipids that impose stress by causing reactive oxygen species (ROS) production, organelle damage and dysfunction, and inflammatory signaling, which, in turn, promote fibrosis via activating stellate cells [4,8,9,10,11]. As this is a pivotal point for adverse effects and a risk factor for mortality and severe comorbidities [5,12], alleviating or resolving lipid-induced stress in the liver may be critical to improve outcomes for patients with MASH.

One strategy to reduce hepatic lipid stress is by enhancing endogenous defense programs; however, which can be targeted to accomplish this feat is not clear. Here, we focus on the homologous and ubiquitously expressed transcription factors, nuclear factor erythroid 2-related factor-1 (Nrf1) and -2 (Nrf2), which are known to regulate expression of cytoprotective defenses [13,14]. Genes that Nrf1 regulates in the liver include those involved in the oxidative stress response (e.g., *Gclc*, *Gclm*, and *Gstm1*), proteostasis (e.g., *Vcp*, *Psma1*, and *Psmc2*), and lipid metabolism (e.g., *Lipin1*, *Ppargc1b*, and *Cd36*) [15,16,17,18,19,20,21]. Genes that Nrf2 regulates in the liver include the same oxidative stress response genes and many more (e.g., *Nqo1*, *Ho1*, and *Gsta3*), as well as those involved in lipid metabolism (e.g., *Ces1g*, *Me1*, and *Abcg8*) [15,17,20,22,23]. Regulation of these gene programs in the liver may promote protection against MASH. Indeed, actions by Nrf1 and Nrf2, determined by us and others using gene-deficient mice, have been shown to affect cholesterol, triglyceride, and glucose metabolism and to defend against ROS, proteotoxicity, and impaired autophagy [15,16,17,18,19,20,21,22,23,24,25,26,27]. Likewise, gene deficiency has been linked to steatohepatitis in mice [16,18,19,20,21,25,26], consistent with the effect of polymorphisms linked to the genes encoding NRF1 (*NFE2L1*) and NRF2 (*NFE2L2*) in people with obesity [28]. Thus, enhancing Nrf1 and/or Nrf2 activity in the liver may be important for mitigating MASH and preventing liver disease progression.

While there is therapeutic potential in targeting these factors, there are incompletely understood aspects regarding similarities and differences for their roles in liver health and disease. One known difference is that newly synthesized Nrf1 is anchored to the endoplasmic reticulum, undergoes Vcp/p97-dependent turnover by the proteasome, and is sensitive to insufficient proteasome activity [14,29,30,31], whereas newly synthesized Nrf2 is localized to the cytosol, forms a heterodimeric complex with kelch-like ECH-associated protein 1, is sensitive to ROS, and can be regulated by the ubiquitin-binding adaptor molecule p62, also known as Sqstm1, which has a major role in HCC development [13,32,33,34,35]. Another is that while they have common targets, they also have distinct gene targets [17,20,36,37,38,39], and there are similar and differing mechanisms by which they control transcription [13,14,40]. For example, both Nrf1 and Nrf2 have been reported to interact with CREB-binding protein and p300 to regulate transcription [40,41,42], whereas transcriptional actions by Nrf1, but apparently not Nrf2, are modulated by nuclei-localized O-linked N-acetylglucosamine transferase [43], and Nrf2, but apparently not Nrf1, is modulated by the Mediator complex [44]. Despite being capable of recognizing the same antioxidant response elements (ARE) in the genome [13,14,40], these differences may render distinct functions. Indeed, we recently identified hepatic Nrf1 and Nrf2 binding to and regulation of several distinct and overlapping genes in the liver of mice [20], and showed this was important for protecting against hepatic cholesterol overload. The most important difference for the current study, however, is regarding a discrepant role they may play in HCC. Nrf1 has been found to repress tumor growth in HCC cell models [45,46,47] and in aged mice with hepatic Nrf1 deficiency [21]. In contrast, while transient pharmacological induction of Nrf2 can improve liver health [48], the role of Nrf2 on HCC formation in transgenic mice has been conflicting [32,35,49]. Specifically, while induction of Nrf2 protects against oxidative damage, chronic induction of Nrf2 by p62 has been shown to promote hepatomegaly and underlie p62-induced HCC [32,33,34,35]. Consistent with this, humans with Nrf2 gain-of-function mutations have a higher HCC incidence than the general population [50,51,52]. Hence, while Nrf1 activity seems to be protective throughout MASLD stages, there appears to be a ‘double-edged sword’ effect for Nrf2 dictated by the mode and duration of activation. Therefore, further investigation is needed regarding the role of Nrf1 and Nrf2 in MASH and its progression to gain sufficient clarity for strategic development of appropriate therapies.

Recently, we demonstrated that Nrf1 and Nrf2 complementarily regulate the expression of hepatocyte defenses to protect against the stress of MASH-associated hepatic cholesterol overload [20]. High fat, fructose, and cholesterol (HFFC) diet-fed mice with a coinciding 1 to 3 weeks of hepatocyte deficiency for Nrf1 and Nrf2, but not either alone, exhibited severe MASH due to increased hepatic lipid storage, altered bile acid metabolism, and oxidative damage, whereas Nrf1 deficiency alone was sufficient to increase hepatic inflammation. Moreover, Nrf1 and Nrf2 were each required for 2-week treatment with the Nrf2-activating drug bardoxolone to reduce steatosis and inflammation in mice chronically fed the MASH-inducing HFFC diet for 24 weeks, and combining this with hepatocyte overexpression of human NRF1 potentiated such effects in mice fed an HFFC diet for 16 weeks. This indicated that enhancing the actions of both Nrf1 and Nrf2 in hepatocytes can reduce MASH. However, the study durations were insufficient to evaluate the impact hepatocyte Nrf1 and Nrf2 activity may have on more severe stages of MASLD, such as cirrhosis and HCC. Here, using HFFC diet-fed conditions lasting up to 52 weeks in duration or combining an HFFC diet with the liver fibrosis-inducing agent carbon tetrachloride and modulating the activity of Nrf1 and/or Nrf2 in differing disease stages, we investigate the hypothesis that Nrf1 and Nrf2 synergistically alleviate MASH and fibrosis and prevent HCC. Our first objective was to examine the effect of hepatic Nrf1, Nrf2, and combined deficiency for increasing periods of time (~7 days, ~7 weeks, and ~35 weeks) on MASH progression and HCC development in mice fed an HFFC diet for 24 or 52 weeks. Our second objective was to examine the effect of inducing hepatic Nrf1, Nrf2, or both on alleviating liver fibrosis in mice fed an HFFC diet for 24 weeks while also being exposed to the liver fibrosis-inducing agent, carbon tetrachloride, for the first 15 weeks. Our results confirm beneficial effects of the Nrf2 inducer bardoxolone to alleviate liver inflammation and fibrosis, establish a robust synergistic role for Nrf1 and Nrf2 to reduce hepatic lipid storage, and demonstrate that hepatocyte Nrf1 plays a greater physiological role than Nrf2 in preventing HCC.

## 2. Results

### 2.1. Effect of HFFC Diet on Liver in Mice with Flox Alleles for Nrf1, Nrf2, or Both

Our initial goal was to investigate the impact of hepatocyte deficiency for Nrf1 (*Nfe2l1*), Nrf2 (*Nfe2l1*), or both on the progression of diet-induced MASH, using *Nfe2l1^flox/flox^* mice, *Nfe2l2^flox/flox^* mice, and *Nfe2l1^flox/flox^*; *Nfe2l2^flox/flox^* mice, respectively, as described previously [20,24,27]. While there are several dietary approaches [8,53,54] with each having advantages and limitations, we selected to use chronic feeding with an HFFC diet, which has been shown to induce several histological, transcriptional, and metabolic features translationally relevant to MASH [55,56,57,58,59,60,61]. Cohorts of male and female mice from each line were fed a 1% cholesterol-containing HFFC diet or a control diet for 24 weeks. At either week 16 or 22, these mice were infected with adeno-associated virus exhibiting hepatocyte expression of green fluorescent protein (AAV-GFP) or Cre recombinase (AAV-CRE). As we have shown [20,24,27], AAV-CRE causes a hepatocyte-specific loss-of-function effect for the respective flox gene whereas AAV-GFP serves as a control. At week 24, tissues were collected for assessment. With these experiments, we assessed the effect of HFFC compared to control diet as well as the impact of hepatic gene deficiency for a short term of ~7 days (i.e., infected at week 22) or prolonged duration of ~7 weeks (i.e., infected at week 16).

**Figure 1:**
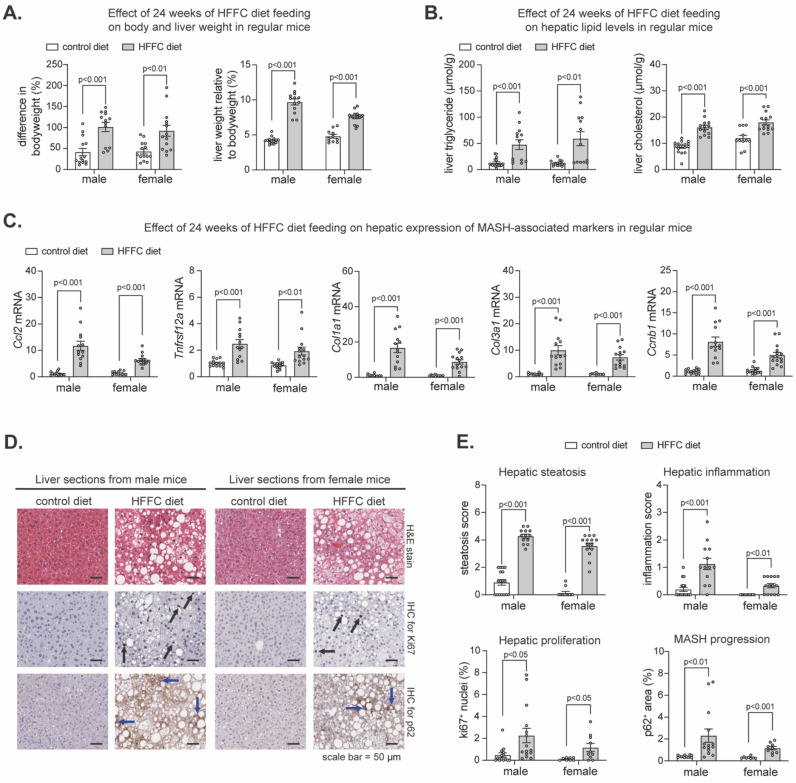
HFFC diet promotes weight gain and MASH. Mice fed control diet or HFFC diet for 24 weeks. (**A**,**B**) % change in relative body weight and % liver-to-body weight ratio (**A**) and levels of triglyceride and cholesterol in liver (**B**), comparing diets (n = 11–17). (**C**) Liver qPCR for indicated gene expression, normalized by *36b4* (n = 11–15). (**D**) Liver sections were stained with hematoxylin and eosin (H&E) or underwent immunohistochemistry (IHC) with antibody-detecting ki67 or p62, with scale in panel. (**E**) Steatosis and inflammation in liver determined using H&E-stained sections, and % of ki67 positive cells and % area of p62 determined using IHC (n = 8–17). Data in (**A**–**C**,**E**) are mean ± standard error of the mean, with individual data points shown. *p*-value was determined by multiple unpaired *t*-tests, with adjustment for multiple comparisons. In (**D**), black arrows indicate Ki67^+^ nuclei and blue arrows indicate p62^+^ area.

To determine the extent to which the flox lines were prone to developing diet-induced MASH, we pooled them and compared MASH-related parameters in AAV-GFP-infected mice fed an HFFC diet to AAV-GFP-infected mice fed a control diet. As expected, chronic HFFC diet increased the body weight and liver-to-body weight ratio in males and females (Figure 1A), coinciding with increased hepatic triglyceride and cholesterol (Figure 1B). Likewise, transcriptional analyses of livers using quantitative polymerase chain reaction (qPCR) revealed HFFC diet intake increased expression of MASH markers involved in liver inflammation (*Ccl2, Tnfrsf12a*), fibrogenesis (*Col1a1, Col3a1*) and cell proliferation (*Ccnb1*) (Figure 1C). To further elucidate the effect of HFFC diet on MASH development, histological examination of liver sections was performed (Figure 1D,E). We used hematoxylin and eosin (H&E) staining to assess steatosis and inflammation, immunohistochemical (IHC) staining of Ki-67 to assess hepatocyte proliferation [62], and IHC staining of p62 as a marker of liver damage, chronic steatohepatitis, and risk for hepatocellular carcinoma (HCC) onset and progression [34,35,63,64]. Compared to control, HFFC diet increased hepatic steatosis and inflammation as well as proliferation and p62 accumulation (Figure 1D,E). Thus, consistent with others [55], chronic HFFC diet intake induced MASH within a 24-week time frame, confirming that using this diet on these mouse lines is suitable for investigating the role of Nrf1 and Nrf2 in the progression of MASH to cirrhosis and HCC.

### 2.2. Effect of Hepatocyte Deficiency for Nrf1, Nrf2, or Both in Mice Chronically Fed HFFC Diet

**Figure 2:**
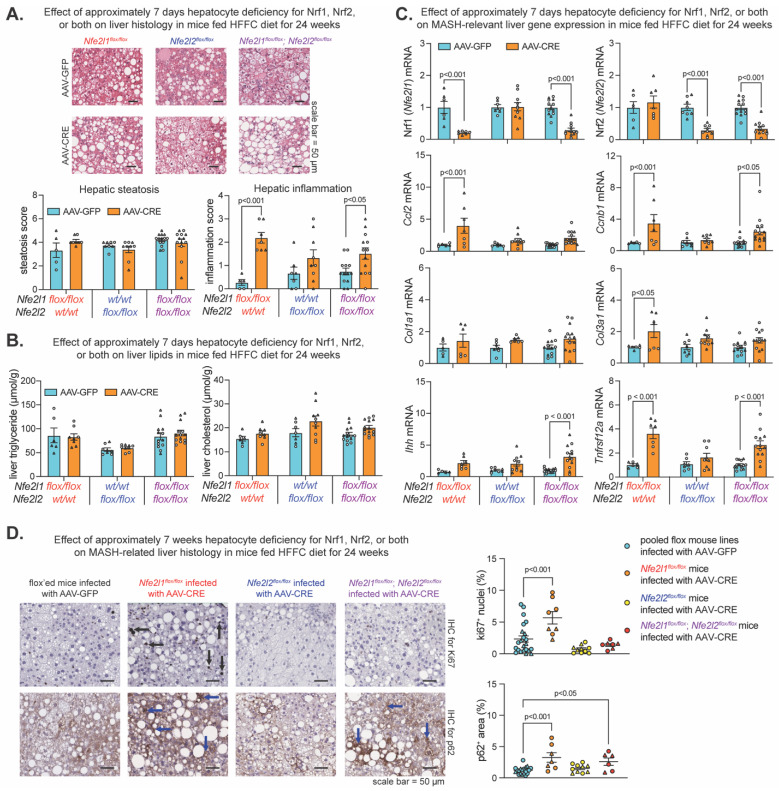
Effect of hepatocyte deficiency for Nrf1, Nrf2, or both in mice chronically fed HFFC diet. Mice fed HFFC diet for 24 weeks. In (**A**–**C**), mice were infected with indicated virus on week 22. In (**D**), mice were infected on week 16. (**A**) Liver sections stained with hematoxylin and eosin, with scale indicated, and steatosis and inflammation (n = 5–14). (**B**) Levels of triglyceride and cholesterol in liver (n = 6–14). (**C**) qPCR analysis for indicated gene expression, normalized by *36b4* (n = 5–14). (**D**) Representative sections for immunohistochemistry (IHC) with antibody-detecting ki67 or p62, with scale indicated, and % of ki67 positive cells and % area of p62 (n = 6–23). Data are mean ± standard error of the mean, with individual data points shown (males = circles; females = triangle). In (**A**–**C**), *p*-value was determined by two-way analysis of variance, with Sidak post-test. In (**D**), *p*-value was determined by one-way analysis of variance, with Dunnett post-test. In (**D**), black arrows indicate Ki67^+^ nuclei and blue arrows indicate p62^+^ area.

We have shown short-term (i.e., 10–21 days) hepatocyte Nrf1 and Nrf2 deficiency, but not single gene deficiency, results in hepatic lipid accumulation, oxidative damage, and inflammation in mice fed an HFFC diet for only 17–28 days [20]. However, the effect on lipid levels did not occur in short-term gene-deficient mice that already had chronic HFFC diet-induced MASH, while other aspects related to liver inflammation did still occur. Here, we investigated the impact of hepatocyte Nrf1, Nrf2, or combined deficiency for ~7 days or for ~7 weeks on MASH progression. Using the long-term diet experimental model described above and, in this case, pooling male and female samples of each flox line, we compared MASH-related parameters in AAV-CRE-infected mice fed an HFFC diet to AAV-GFP-infected mice fed an HFFC diet (Figure 2), as well as the AAV-CRE-infected mice fed a control diet to AAV-GFP-infected mice fed a control diet (Figure S1).

Compared to the respective control, HFFC diet-fed mice with ~7 days hepatocyte deficiency for Nrf1, Nrf2, or both had no difference in the level of steatosis and hepatic lipids (Figure 2A,B). However, Nrf1 deficiency increased liver inflammation (Figure 2A). As expected, there was reduced mRNA of Nrf1 (*Nfe2l1*) and Nrf2 (*Nfe2l2*) in respective AAV-CRE-infected flox lines, in which case, hepatic Nrf1 deficiency increased expression of MASH-related genes *Ccl2*, *Ccnb1*, *Col3a1*, and *Tnfrsf12a* (Figure 2C). Likewise, combined deficiency increased liver inflammation (Figure 2A) and expression of *Ccnb1*, *Col3a1*, *Ihh*, and *Tnfrsf12a* (Figure 2C). In contrast, Nrf2 deficiency had no effect. In control diet fed mice, Nrf2 deficiency was nonconsequential, while Nrf1 and combined deficiency exacerbated the effect, as demonstrated by hepatic steatosis as well as significant or trending increases in hepatic triglyceride and expression of *Ccl2*, *Ccnb1*, *Col1a1*, *Col3a1*, *Ihh*, and *Tnfrsf12a* (Figure S1A–C). Also as expected, there was reduced mRNA of Nrf1 (Nfe2l1) and Nrf2 (Nfe2l2) in respective flox lines infected with AAV-CRE (Figure S1C).

We then evaluated whether prolonged ~7-week deficiency for Nrf1, Nrf2, or both resulted in signs of major adverse liver outcomes reflecting the progression of MASH to HCC. Using IHC, we stained for Ki67 to assess hepatocyte proliferation, and p62 to assess liver damage and HCC risk. As expected, Nrf2 deficiency had no effect, but interestingly, while mice with Nrf1 deficiency had increased Ki67 positive nuclei irrespective of being fed an HFFC or control diet, this did not occur in mice with combined deficiency (Figure 2D and Figure S1D). Moreover, Nrf1 deficiency increased p62 positive nuclei, whereas this occurred only for mice with combined deficiency when fed an HFFC diet (Figure 2D and Figure S1D). Taken together, results from the ~7-day deletion model demonstrate a predominant physiologic role for Nrf1 in counteracting liver inflammation, whereas results from the ~7-week deletion model suggest Nrf1 counteracts MASH progression to HCC, possibly via influencing pro-tumorigenic actions of Nrf2.

### 2.3. Nrf1 Deficiency Enhances Liver Tumorigenesis in Male Mice with MASH

**Figure 3:**
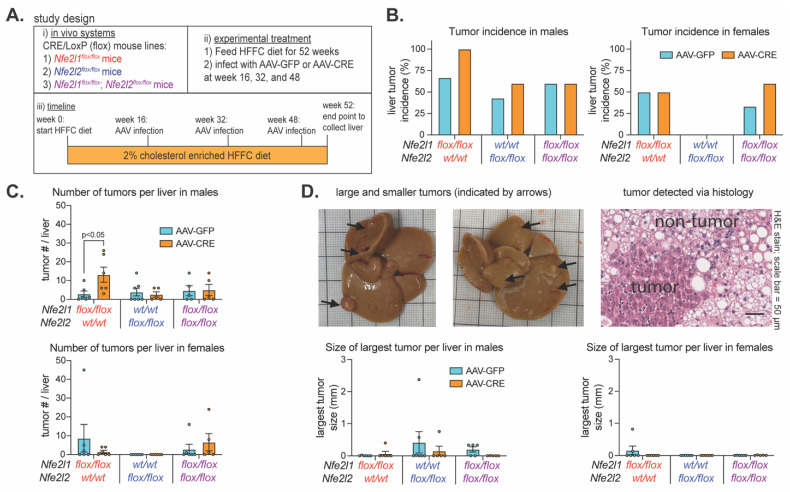
Effect of hepatocyte deficiency for Nrf1, Nrf2, or both on liver tumor development. (**A**) Study design showing that mice were fed HFFC diet with 2% cholesterol for 52 weeks and infected on weeks 16, 32, and 48 with indicated virus. Liver tumor analysis was performed at the endpoint. (**B**,**C**) % incidence of liver tumors (**B**) and the number of tumors per liver (**C**) in males (n = 5–7) and females (n = 5–6). (**D**) Volume of largest liver tumor in males (n = 5–7) and females (n = 5–6) and representative tumor images (black arrows indicate tumors). Data are mean ± standard error of the mean, with individual data points shown. The *p*-value was determined by two-way analysis of variance, with Sidak post-test.

As Nrf1 deficiency for ~7 days to ~7 weeks corresponded with signature features of liver disease progression and this was distinct from Nrf2, we next examined their roles in protecting against MASH progression to liver tumorigenesis. Male and female *Nfe2l1^flox/flox^* mice, *Nfe2l2^flox/flox^* mice, and *Nfe2l1^flox/flox^*; *Nfe2l2^flox/flox^* were fed a 2% cholesterol-enriched HFFC diet for 52 weeks. On weeks 16, 32, and 48, mice were infected with AAV-CRE to induce and maintain hepatocyte deficiency of Nrf1, Nrf2, or both, respectively (Figure 3A). Control mice were infected with AAV-GFP. Thus, we examined whether ~35 weeks of hepatocyte deficiency in mice with pre-existing and persisting MASH impacted HCC tumor onset and growth.

At the study endpoint, livers were collected to assess the tumor incidence and abundance. Males and females were analyzed separately due to apparent sex-dependent differences in incidence, as there was an average of 56% incidence in AAV-GFP-infected male mice and 27% in AAV-GFP-infected female mice (Figure 3B). Tumor incidence in female mice with hepatocyte deficiency for Nrf1, Nrf2, or both was not obviously different (i.e., <30%) than controls, and this was also the case for male mice with Nrf2 deficiency and those with combined deficiency (Figure 3C). In contrast, incidence in males with Nrf1 deficiency was 100% and >30% greater than controls, and this also corresponded with more tumors per liver (Figure 3C). Interestingly though, number of tumors per liver was similar compared to control in males with combined deficiency, indicating that tumorigenic effects of Nrf1 deficiency depended on Nrf2 activity. However, the size of the largest tumors was not greater in hepatocyte Nrf1 deficient liver (Figure 3D), indicating tumorigenic effects were due to increased tumor initiation, not tumor growth. Taken together, these results suggest that physiologic Nrf1 protects against MASH progression to HCC by suppressing tumor initiation, and that this may involve functional interactions with Nrf2 activity.

### 2.4. Effect of Bardoxolone and Nrf1 Overexpression on MASH-Linked Fibrosis

**Figure 4:**
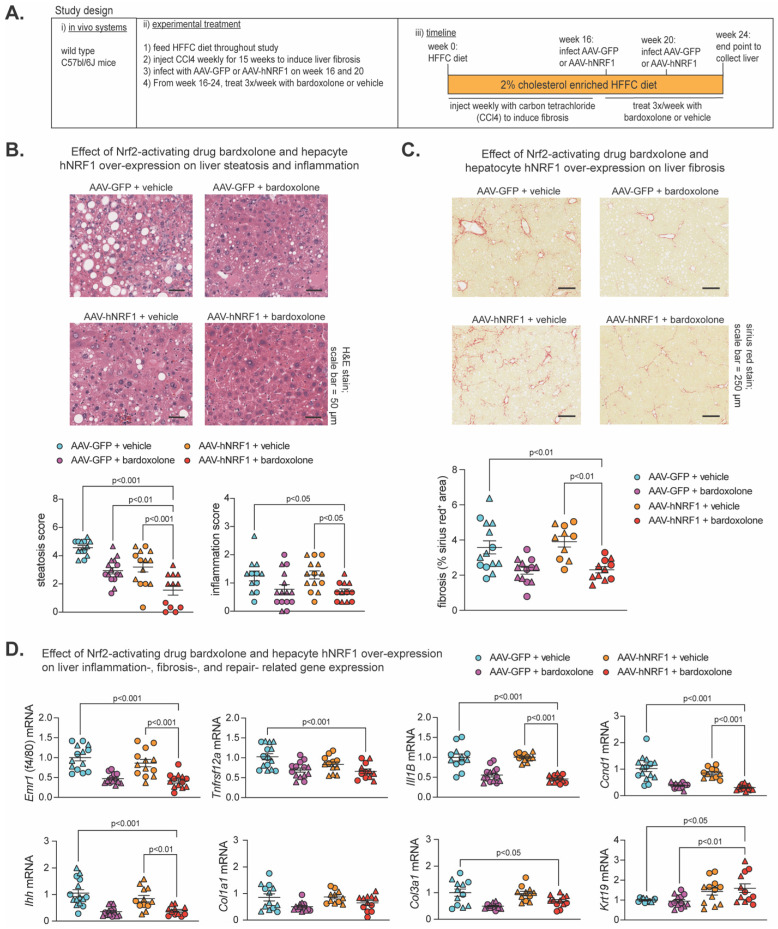
Combined effect of Nrf2-inducing drug bardoxolone and Nrf1 overexpression on MASH-linked fibrosis. (**A**) Study design showing C57bl/6J mice that were fed HFFC diet with 2% cholesterol for 24 weeks. Mice were injected with carbon tetrachloride once per week from week 0–15 to induce liver fibrosis. On weeks 16–24, mice were treated as indicated with modulators of Nrf1 and Nrf2 activity. Liver analysis was performed at the endpoint. (**B**) Liver sections stained with hematoxylin and eosin, with scale indicated in panel, and corresponding score for steatosis and inflammation in liver (n = 12–15). (**C**) Liver sections stained with fibrosis detecting sirius red, with scale indicated in panel, and corresponding % sirius red^+^ area (n = 10–14). (**D**) Liver qPCR analysis for indicated gene expression, normalized by ribosomal protein *36b4* (n = 11–15). Data are mean ± standard error of the mean, with individual data points shown (males = circles; females = triangles). The *p*-value was determined by one-way analysis of variance, with Dunnett post-test.

Previously [20], we showed both Nrf1 and Nrf2 were required for beneficial effects of 2-week treatment with the Nrf2-activating drug bardoxolone on MASH in 24-week HFFC diet-fed mice, and combining 1-week bardoxolone treatment with hepatocyte overexpression of human NRF1 (hNRF1) exhibited a partial synergistic effect in mice fed an HFFC diet for 16 weeks. Using similar methods to induce Nrf1 and Nrf2, here we investigated whether more prolonged induction of Nrf1, Nrf2, or both may alleviate MASH and fibrosis in a MASH-linked fibrosis model. As illustrated in Figure 4A, male and female C57bl/6J mice were fed a 2% cholesterol-enriched HFFC diet for 24 weeks. To induce liver fibrosis, mice were injected with the hepatocyte-damaging agent carbon tetrachloride [65,66,67,68] once per week for the first 15 weeks. At week 16 and 20, mice were infected with AAV-hNRF1 to induce and maintain increased hepatocyte Nrf1 activity or with AAV-GFP, as a control. Also, from week 16 to 24, mice were injected three times per week with bardoxolone or vehicle control. At week 24, liver was collected for analysis of the four groups: AAV-GFP + vehicle, AAV-GFP + bardoxolone, AAV-hNRF1 + vehicle, and AAV-hNRF1 + bardoxolone.

To ensure each treatment had the intended effect, we assessed expression of Nrf1 and Nrf2 and their target genes, *Psmc2* and *Gstm1*, respectively, which we demonstrated previously [20]. Compared to AAV-GFP + vehicle, livers expressing hNRF1 had increased NRF1 level and *Psmc2* expression, whereas livers treated with bardoxolone had increased Nrf2 and *Gstm1* expression (Figure S2A,B), thus validating the model. Then, we performed histological analysis via H&E staining to assess steatosis and inflammation, as well as sirius red staining and IHC for hydroxyproline to assess liver fibrosis (Figure 4B,C and Figure S2C). Compared to AAV-GFP + vehicle, AAV-hNRF1 + bardoxolone reduced steatosis, and this was greater than AAV-GFP + bardoxolone and AAV-hNRF1 + vehicle. In contrast, AAV-hNRF1 + bardoxolone reduced liver inflammation and fibrosis (Figure 4B,C and Figure S2C) compared to AAV-GFP + vehicle and AAV-hNRF1 + vehicle, but not AAV-GFP + bardoxolone.

Interestingly, while evaluating H&E-stained sections, the AAV-hNRF1 + bardoxolone group had a noticeable population of oval-shaped cells reminiscent of liver progenitor cells (LPC), which are known to sequester in the canals of Hering and to differentiate into functional hepatocytes in conditions of chronic liver injury [69,70,71]. As oval cells have been found to repopulate liver with functionally active hepatocytes following chronic liver injury [72], we sought to quantify whether this population was affected by Nrf1 and Nrf2 activity. To do this, we measured the expression of cytokeratin 19 (*Krt19*), a marker of LPC activation, while also measuring known markers of liver inflammation (*Emr1*, *Tnfrsf12a*, *Il1B*), fibrosis (*Ihh*, *Col1a1*, and *Col3a1*), and proliferation (*Ccnd1*). Compared to AAV-GFP + vehicle control, AAV-hNRF1 + bardoxolone reduced expression of genes involved in liver inflammation, proliferation, and some involved in fibrosis, which in some cases were also reduced compared to AAV-hNRF1 + vehicle but not to AAV-GFP + bardoxolone. In contrast, AAV-hNRF1 + bardoxolone increased *Krt19* expression, and this was also increased compared to AAV-GFP + bardoxolone but not to AAV-hNRF1 + vehicle. Taken together, these results indicate that the combined induction of Nrf1 and Nrf2 synergistically reduces hepatic lipid stores in MASH-linked fibrosis and that the Nrf2-inducing drug bardoxolone is sufficient to reduce liver inflammation, proliferation, and fibrosis, whereas actions by Nrf1 may enhance the repair and functional cell repopulation by LPCs in chronically damaged liver.

## 3. Discussion

MASH and its progression to cirrhosis and HCC is a global health burden [2,4,6,7,12]. Effective therapeutic strategies are in need, but this is hindered by an incomplete understanding of how to counteract the pathologic mechanisms. Stress caused by hepatotoxic lipid accumulation is recognized as a central factor [4,8,9,10,11], and we propose enhancing endogenous defense programs controlled by transcription factors Nrf1 and Nrf2 may alleviate this stress to mitigate MASH and its progression. Here, we explored this possibility using a validated model of diet-induced MASH on transgenic mice amenable to adult-onset loss-of-function for hepatocyte Nrf1, Nrf2, or both and by enhancing the actions of Nrf1, Nrf2, or both in wild-type mice with MASH-linked fibrosis. Our results show hepatic Nrf1 is important for preventing MASH-linked hepatocyte proliferation, liver inflammation, and HCC, that actions by Nrf1 and Nrf2 synergistically reduce hepatic lipid storage, and that the Nrf2-inducing drug bardoxolone alleviates liver inflammation and fibrosis. Altering the activity of hepatic Nrf1, Nrf2, or both had a largely similar impact on males and females, except that the magnitude of the effect was in some cases more dramatic in one sex and tumor incidence was only different in hepatic Nrf1-deficient males. The reasons for such differences are uncertain but may relate to previously recognized sexual dimorphism driven by estrogen receptor α [73]. Overall, the results here are consistent with previous studies of the individual role of Nrf1 and Nrf2 on liver steatosis and steatohepatitis using embryonic null mice or transgenic promoter-driven Cre recombinase mice crossed with Nrf1 flox mice [15,16,17,18,19,21,22,23,25,26]. But to our knowledge, our previous study has been the only one to systematically investigate the combined roles of hepatic Nrf1 and Nrf2 on stress defenses counteracting MASH [20], and this study is the only one to assess the combined roles of hepatic Nrf1 and Nrf2 on MASH-linked liver fibrosis and HCC.

Consistent with work by us and others [15,18,20,21,25,26], we found physiologic actions by hepatocyte Nrf1 were critical for mitigating liver inflammation. Some effects were independent of MASH-inducing diet as they occurred and were, in many cases, even greater in mice fed a control diet. Though we did not delineate the mechanism, we speculate this Nrf1-selective baseline effect on inflammation may be due to its unique effects on proteostasis and proteasome activity [16] or some other gene program that it, not Nrf2, can control. For that matter, we previously combined multi-systematic analyses to identify Nrf1- and Nrf2-specific and -overlapping regulatory gene networks, which revealed hundreds of direct gene targets and thousands of regulated genes [20]. Future work may consider exploring direct connections between hepatic Nrf1, gene programming, and liver inflammation as these findings reinforce that Nrf1 plays a major role in liver homeostasis. On the other hand, physiologic actions by hepatocyte Nrf2 were not critical and, instead, may have even contributed to the pathology that arises in Nrf1 deficiency, such as liver tumor development, though we did not provide definitive evidence to that effect. Still, this is a counterintuitive concept, as the transient induction of Nrf2 is generally protective against cellular insult [13], but it has been clearly shown that dysregulated, chronic induction of Nrf2 by p62 is pathological in liver [33] and that Nrf2 and p62 in liver coordinately promote HCC [32,35]. Moreover, Nrf2 gain-of-function mutations have been linked to HCC in humans [8,50,52], which reinforces that there is a significant role for Nrf2 in liver health and disease that requires careful consideration. MASH-induced HCC is a major challenge and still poorly understood [8], and a role for Nrf1 in preventing this process is possible. Xu et al. [21] found liver-specific Nrf1 deletion in utero caused liver tumors when mice reached the adult age of 1 year. Here, we used a similar approach except that hepatocyte Nrf1 was not deleted until mice were well into adulthood and had been fed an HFFC diet for at least 16 weeks. Tumor number was elevated in male mice with Nrf1 deficiency, and interestingly, this effect was lost in mice deficient for Nrf1 and Nrf2. Thus, Nrf1 may be able to mitigate pro-tumorigenic effects of chronic Nrf2 activation that have been identified by others [32,35,50,51,52].

Interestingly, while loss of physiologic Nrf2 activity had little effect, the opposite occurred when using the Nrf2-activating agent bardoxolone. Combined induction of Nrf2 via bardoxolone and Nrf1 via hNRF1 expression reduced steatosis, inflammation, proliferation, and fibrosis. Most of these effects were attributable mainly to bardoxolone, as only reduced steatosis was amplified by combined treatment. Caution with interpretation is necessary, as bardoxolone could elicit these effects by impacting non-hepatic tissue. Still, these results raise two interesting questions. First, what is the mechanism by which bardoxolone and AAV-hNRF1 synergize to reduce hepatic lipids? Second, assuming actions of bardoxolone were due to hepatocyte Nrf2, what drives the disconnect between an apparently dispensable physiologic Nrf2 with an apparently potent Nrf2-inducing drug. Future work should aim to address the underlying mechanism of this discrepancy. Answers to these questions may be important, given that bardoxolone was initially developed as a therapeutic meant to reduce inflammation-driven cancer [74]. However, there are limitations of the present study we must recognize. One is that we employed AAV infection, which may alter the trajectory of MASH-induced liver fibrosis and HCC. Likewise, the AAV model system may have limited utility in long-term studies, since if any hepatocytes undergo apoptosis, the replacing hepatocyte will not have the gene alteration. For this reason, we performed repeat infections of AAV-CRE during our 52-week study. Another important limitation is that we investigated MASH progression in mice and there is not yet a clear consensus and understanding of how to effectively translate studies on HCC in mice to HCC in humans [8,53,54].

Consistent with our previous work [20], functional Nrf1 was important for preventing the accumulation of the liver damage and HCC marker p62, but it is unknown how this effect develops. Another interesting finding was that Nrf1, and not Nrf2, induction using AAV-hNRF1 increased expression of the LPC marker *Krt19*, and that LPCs were abundant in AAV-hNRF1 conditions. This may indicate Nrf1 has a role in liver repair and in resisting liver damage. Investigating Nrf1 in other conditions of experimental liver damage may provide more clarity. In conclusion, we identified distinct and complementary roles for hepatocyte Nrf1 and Nrf2 in protecting against MASLD progression and in promoting hepatocyte regeneration in liver with MASH-linked fibrosis. Hence, therapies targeting defense programs controlled by Nrf1 and Nrf2 may prove to be an effective approach for improving the outcomes of patients with MASLD.

## 4. Materials and Methods

### 4.1. Animals Used in Study

Mouse handling, husbandry, and experiments were performed with approval from the University of Saskatchewan’s Animal Care Committee, according to animal use protocol number AUP20180090. Ethical considerations follow the ARRIVE guidelines. In loss-of-function studies, male and female mice on C57bl/6J background were housed at 21 °C on a 12 h light/dark cycle and provided ad libitum access to food and water. Mice were fed a control diet containing 10% fat (lard and soybean oil), 10% sucrose, 0% fructose, and 0% cholesterol (Research diets, New Brunswick, NJ, USA; catalog# D09100304) or MASH-inducing HFFC diet containing 40% fat (75% palm oil/11% lard/14% soybean oil), 10% sucrose, 20% fructose and 1% or 2% cholesterol (Research diets, New Brunswick, NJ, USA; catalog# D19021910 or D09100310), as indicated in the text and figures. Mice with flox alleles for genes encoding Nrf1 (*Nfe2l1^flox/flox^*), Nrf2 (*Nfe2l2^flox/flox^*), or both (*Nfe2l1^flox/flox^*; *Nfe2l2^flox/flox^*) were generated as in previous studies [18,20,24,27]. Diet feeding ranged from 24 to 52 weeks, as indicated. To delete Nrf1, Nrf2, or both in hepatocytes, recombination of flox alleles to remove gene elements was performed by retroorbital infection of mice, while under isoflurane anesthesia, with 2.0 × 10^11^ particles of liver-targeting serotype 8 adeno-associated virus expressing Cre recombinase via hepatocyte-specific thyroxine-binding globulin promoter (AAV-CRE), as in previous studies [20,24,27]. Controls received virus expressing green fluorescent protein (AAV-GFP). AAV-CRE (AAV8.TBG.PI.Cre.rBG) and AAV-GFP (AAV8.TBG.PI.eGFP.WPRE.bGH) viruses were acquired from the University of Pennsylvania Vector Biocore, Philadelphia, PA, USA.

In the gain-of-function study, C57bl/6J mice were purchased from The Jackson Laboratory (Bar Harbor, ME, USA; RRID: IMSR_JAX:000664) and fed an HFFC diet throughout. Liver fibrosis was induced using a method similar to Tsuchida et al. [65]. Liver toxin carbon tetrachloride (CCL4; Sigma-Aldrich, Saint Louis, MO, USA; catalog# 319961) was diluted 1:10 in corn oil (Sigma-Aldrich, Saint Louis, MO, USA; catalog# C8267) and intraperitoneally (IP) injected to mice at a dose of 0.32 μg/g (0.2 μL/g) body weight once per week for 15 weeks. To enhance hepatocyte Nrf1 activity, mice were injected with the same AAV as above, but encoding for human NRF1 (AAV-hNRF1) expression transcript (NCBI: NM_003204.2) as in a previous study [20]. Littermate controls received virus expressing green fluorescent protein (AAV-GFP). AAV-hNRF1 (AAV8.TBG.hNRF1) was acquired from the University of Pennsylvania Vector Biocore, Philadelphia, PA, USA. To enhance hepatocyte Nrf2 activity, mice were injected IP with 3 mg/kg bardoxolone methyl (bardoxolone) (Selleckchem, Houston, TX, USA; catalog# S6647). Controls received vehicle (10% DMSO in PBS). Altogether, mice were infected with AAV-hNRF1 or AAV-GFP at week 16 and 20, while bardoxolone or vehicle was administered three times a week for 8 weeks, starting at week 16.

### 4.2. Tissue Collection and Liver Histological Analysis

At indicated endpoints, mice were anesthetized via 3% isoflurane at an oxygen flow rate of 1 L/minute. Blood was collected into a 26-gauge 1 mL syringe needle via intracardiac puncture and then placed into a 1.5 mL tube containing ethylenediaminetetraacetic acid (EDTA, 5 mM final concentration) and placed on ice. Later, plasma was separated via 2000 g centrifugation at 4 °C for 10 min. Immediately after collecting blood, the vasculature of anesthetized mice was flushed with 20 mL of phosphate buffered saline that was at room temperature. The liver was isolated and weighed and then divided into portions that were snap-frozen and stored at −80 °C. Another portion of the liver (left lobe) was used for histological analysis. The left lobe was fixed in 10% neutral buffered formalin, embedded in wax, sectioned at 5 µm thickness, and stained with hematoxylin and eosin (H&E) or with agents described below. Images were captured using an Aperio Scanscope CS image analysis system and Aperio Imagescope viewing software (version 12).

To assess levels of steatosis and inflammation of H&E-stained liver sections, a blinded analyst scored three separate fields for each sample, in accordance with guidelines by Liang et al. [75]. To assess fibrosis, the liver was stained with sirius red (Picro Sirius Red Stain from Abcam, Waltham, MA, USA; catalog #ab150681) or with anti-hydroxyproline antibody from Cell Signaling Technology, Danvers, MA, USA (1:200; catalog #73812S). For anti-ki67 staining and anti-p62, paraffin-embedded 5 µm sections were incubated with anti-ki67 antibody (1:1000; D3B5; IHC formulated; catalog #12202S) or anti-p62 antibody (1:250; D6M5X; catalog #23214S) from Cell Signaling Technology, Danvers, MA, USA overnight at 4 °C; then, they were incubated with peroxidase-labeled secondary antibody for 30 min, stained with diaminobenzidine (DAB), and counterstained with hematoxylin. Three fields per sample were visualized and analyzed with ImageJ software (Fiji RRID: SCR_002285, La Jolla, CA, USA) to determine the percentage of stained area.

### 4.3. Analysis of Liver Cholesterol and Triglyceride

As in a previous study [20], 50–100 mg of frozen liver was homogenized in 2 mL hexane: isopropanol (v:v, 3:2). The homogenate was heated to 75 °C for 5 min and centrifuged at 13,000× *g* for 10 min and then lipid-containing supernatant was transferred to a new tube. The supernatant was dried overnight at 55 °C and resuspended in isopropanol: NP-40 (v:v, 9.9:0.1). Triglyceride was measured using Infinity Triglyceride Reagent from ThermoFisher Scientific, Middletown, VA, USA (catalog #TR22421). To measure liver cholesterol, an aliquot of the isopropanol:NP-40 suspension was dried again at 55 °C, and then resuspended in a cholesterol suspension buffer, a component of the Amplex Red Cholesterol Assay Kit from Invitrogen, Middletown, VA, USA (catalog #A12216) used to measure cholesterol.

### 4.4. Gene Expression Analysis

An amount of 25–50 mg of liver was homogenized in TRIzol Reagent ThermoFisher Scientific; catalog #15596018) and RNA isolated according to the manufacturer’s instructions. Complementary DNA (cDNA) was synthesized using 400 ng of RNA substrate via a Maxima H Minus First Strand cDNA Synthesis Kit with dsDNase from ThermoFisher Scientific (catalog #K1672). qPCR was performed on a CFX384 Real-Time PCR Detection System using PowerUpTM SYBRTM Green Master Mix SYBR from ThermoFisher Scientific (catalog #A25742). Gene of interest cycle thresholds were normalized to *ribosomal protein*, *large*, *P0* levels (*Rplp0*; also known as 36B4) by the ΔΔCt (2^−∆∆Ct^) method and displayed as expression levels relative to the experimental control group. The primers used to detect gene expression are described in Supplementary Materials, Table S1.

### 4.5. Tumor Volume Measurement

Tumor dimensions were taken using a Vernier caliper and volume was calculated using the formula: V = (4/3 × (3.14159) × (Length/2) × (Width/2)^2^) as described [76].

### 4.6. Statistical Analysis

Data analysis was performed using GraphPad PRISM (version 9.3.1). Male and female mice results were combined for analysis, unless otherwise indicated in text or figures. Data are presented as mean ± SEM, with individual data points shown as indicated in figures. Significant differences were determined using multiple *t*-test (two-tailed unpaired) with adjustment for multiple comparisons performed using the Holm–Sidak method, one way analysis of variance (ANOVA) or two-way ANOVA, as indicated in figure legends. The significance level was set at *p* < 0.05.

## Data Availability

Data corresponding to figures have been provided in the Mendeley repository (https://data.mendeley.com/datasets/hr4y2pvy8b/1), accessed on 21 July 2024.

## Supplementary Materials

The following supporting information can be downloaded at: https://www.mdpi.com/article/10.3390/ijms25158046/s1.
