# Supplementary material for: Protective Effects of Hepatocyte Stress Defenders, Nrf1 and Nrf2, against MASLD Progression
Source: Int J Mol Sci. 2024 Jul 24;25(15):8046. doi: 10.3390/ijms25158046 (PMC11312428; doi:10.3390/ijms25158046)
Supplement: Supplementary file 1 [file ijms-25-08046-s001.zip › ijms-3090633-supplementary.pdf]

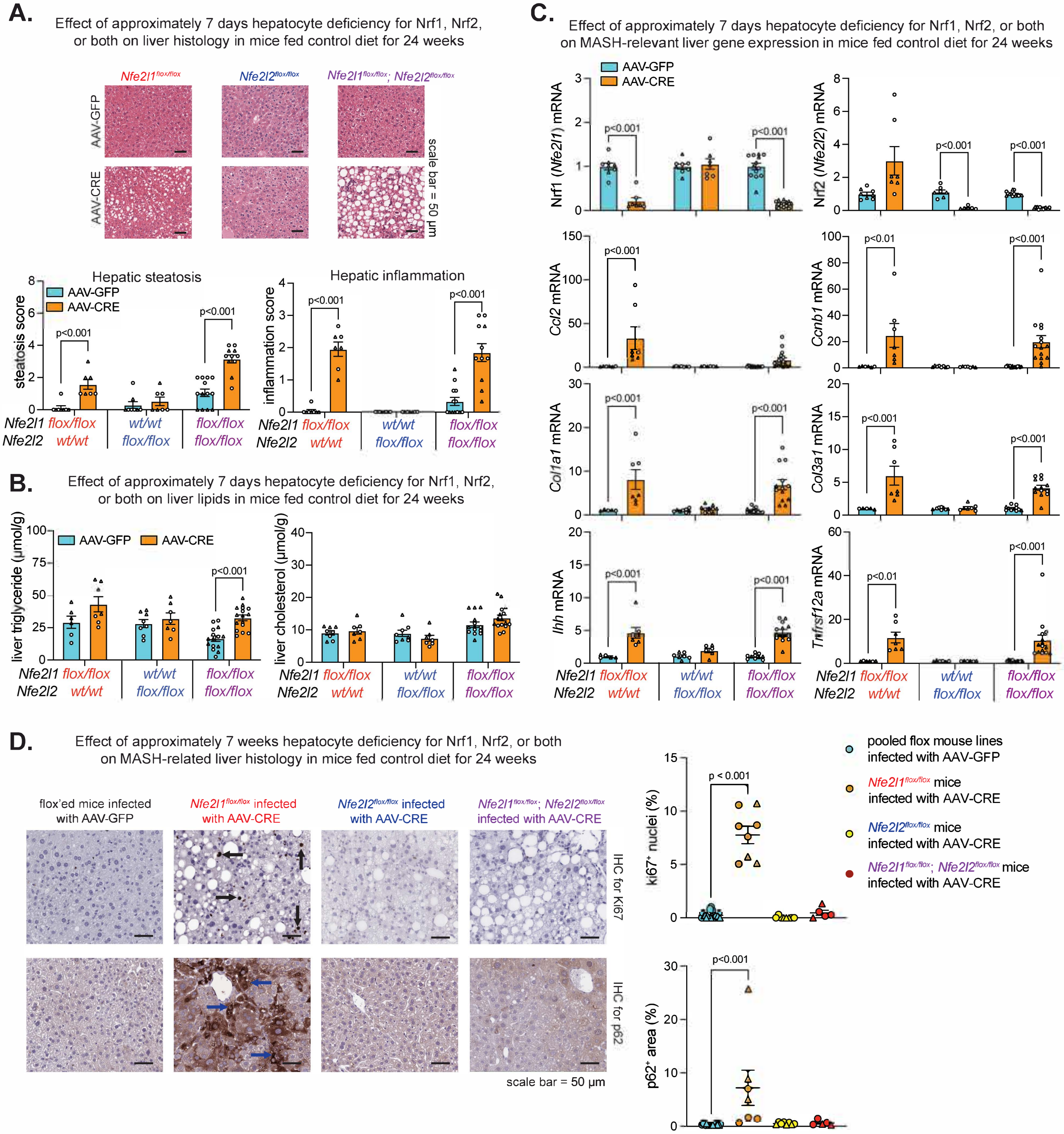


**Supplemental Figure S1. Effect of hepatocyte deficiency for Nrf1, Nrf2, or both in mice chronically fed control diet.** Mice were fed control diet for 24 weeks. In A-C), mice were infected with indicated virus on week 22. In D), mice were infected on week 16. A) Liver sections stained with hematoxylin and eosin, with scale indicated in panel, and steatosis and inflammation in liver (n = 7-13). B) Levels of triglyceride and cholesterol in liver (n = 6-16). C) Liver qPCR analysis for indicated gene expression, normalized by ribosomal protein 36b4 (n = 5-16). D) Representative liver sections that underwent immunohistochemistry (IHC) with antibody detecting ki67 or p62, with scale indicated in panel, and % of ki67 positive cells and % area of p62 (n = 5-20). Data are mean ± standard error of the mean, with individual data points shown (males = circles; females = triangle). In A-C), the p-value was determined by two-way analysis of variance, with Sidak post-test. In D), the p-value was determined by one-way analysis of variance, with Dunnett post-test, and black arrows indicate $Ki67^{+}$ nuclei and blue arrows indicate $p62^{+}$ area.

**A.** Experimental validation that Nrf2-activating drug bardxolone results in increased Nrf2 protein level in the liver, and hepacyte hNRF1 over-expression results in increased NRF1 protein level in the liver

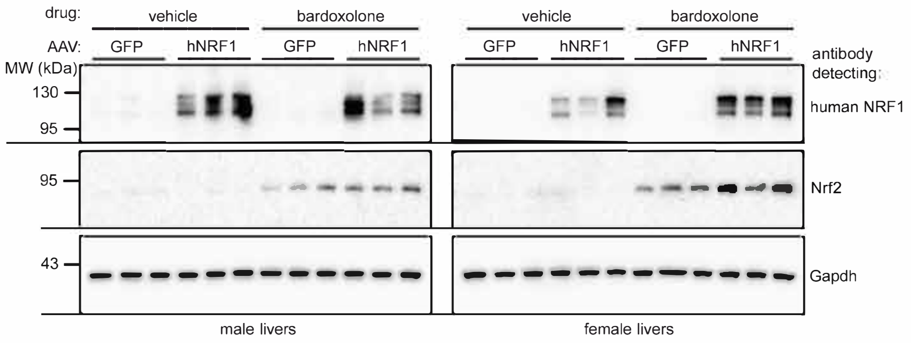


**B.** Experimental validation that Nrf2-activating drug bardxolone induces expresison of Nrf2 target, *Gstm1*, and hepacyte hNRF1 over-expression induces expression of Nrf1 target, *Psmc2*, in the liver

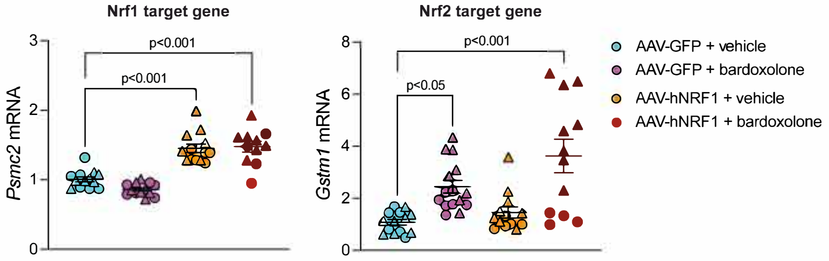


**C.** Effect of Nrf2-activating drug bardxolone and hepatocyte hNRF1 over-expression on liver fibrosis

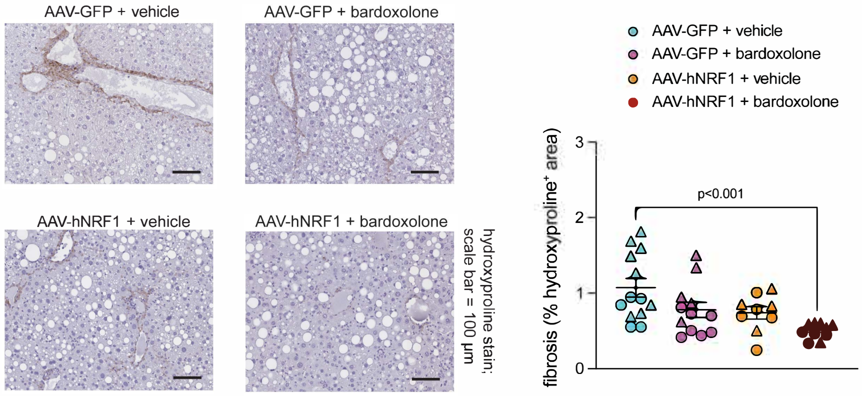


**Supplemental Figure S2. Validating liver induction of Nrf1 via hNRF1 and of Nrf2 via bardoxolone and effect on liver fibrosis.** Corresponding with figure 4 in which C57bl/6J mice were fed HFFC diet with 2% cholesterol for 24 weeks. Mice were injected with carbon tetrachloride once per week from week 0-15 to induce liver fibrosis. On week 16-24, mice were treated as indicated with modulators of Nrf1 and Nrf2 activity. Liver analysis was done at the endpoint. A) Immunoblot for Nrf1 and Nrf2, with Gapdh done as a control of protein loading (n = 3 males; 3 females). B) Liver qPCR analysis for expression of Nrf1 target gene, *Psmc2*, and Nrf2 target gene, *Gstm1*. Expression was normalized by ribosomal protein *36b4* (n = 4-7 males; 7-8 females). C) Liver sections that underwent immunohistochemical detection of fibrosis marker, hydroxyproline, with scale indicated in panel, and corresponding % hydroxyproline positive area (n = 4-7 males; 4-8 females). Data are mean ± standard error of the mean, with individual data points shown (males = circles; females = triangle). The p-value was determined by one-way analysis of variance, with Dunnett post-test.

Supplemental Table S1

| gene name | primer direction | primer sequence |
|---|---|---|
| 36b4 *(Rplp0)* | forward | AGGGCGACCTGGAAGTCC |
| | reverse | CCCACAATGAAGCATTTTGGA |
| *Ccl2* | forward | TTAAAAACCTGGATCGGAACCAA |
| | reverse | GCATTAGCTTCAGATTTACGGGT |
| *Ccnb1* | forward | AAGGTGCCTGTGTGTGAACC |
| | reverse | GTCAGCCCCATCATCTGCG |
| *Ccnd1* | forward | GCGTACCCTGACACCAATCTC |
| | reverse | CTCCTCTTCGCACTTCTGCTC |
| *Col1a1* | forward | TGCTAACGTGGTTCGTGACCGT |
| | reverse | ACATCTTGAGGTCGCGGCATGT |
| *Col13a1* | forward | ACGTAAGCACTGGTGGACAG |
| | reverse | CCGGCTGGAAAGAAGTCTGA |
| *Emr1* | forward | TGACTCACCTTGTGGTCCTAA |
| *(F4/80)* | reverse | CTTCCCAGAATCCAGTCTTTCC |
| *Gstm1* | forward | ATACTGGGATACTGGAACGTCC |
| | reverse | AGTCAGGGTTGTAACAGAGCAT |
| *Ihh* | forward | CTCTTGCCTACAAGCAGTTCA |
| | reverse | CCGTGTTCTCCTCGTCCTT |
| *Il1β* | forward | GCAACTGTTCCTGAACTCAACT |
| | reverse | ATCTTTTGGGGTCCGTCAACT |
| *Krt19* | forward | AGCGTGATCAGCGGTTTTG |
| | reverse | CCTGGTTCTGGCGCTCTATG |
| Nrf1 (*Nfe2l1* ) | forward | GACAAGATCATCAACCTGCCTGTAG |
| | reverse | GCTCACTTCCTCCGGTCCTTTG |
| Nrf2 (*Nfe2l2* ) | forward | CAGCTCAAGGGCACAGTGC |
| | reverse | GTGGCCCAAGTCTTGCTCC |
| *Psmc2* | forward | AGGTGCTGTGTAATCACCGAG |
| | reverse | GGATGGGCTTGTCGTCCTT |
| *Tnfrsf12a* | forward | GTGTTGGGATTCGGCTTGGT |
| | reverse | GTCCATGCACTTGTCGAGGTC |
